# Comparison of child abuse history in patients with and without functional abdominal pain: a case-control study

**DOI:** 10.1186/s12888-020-02675-0

**Published:** 2020-05-24

**Authors:** Seyed Mojtaba Hashemi, Parsa Yousefichaijan, Bahman Salehi, Amir Almasi-Hashiani, Mohammad Rafiei, Sima Zahedi, Esmaeil Khedmati Morasae, Fereshteh Maghsoudlou

**Affiliations:** 1 Department of Pediatric Gastroenterology, Arak University of Medical Sciences, Arak, Iran; 2 Department of Pediatric Nephrology, Arak University of Medical Sciences, Arak, Iran; 3 Department of Psychiatry, Arak University of Medical Sciences, Arak, Iran; 4 Department of Epidemiology, Arak University of Medical Sciences, Arak, Iran; 5 Traditional and Complementary Medicine Research Center, Arak University of Medical Sciences, Arak, Iran; 6 Department of Biostatistics, Arak University of Medical Sciences, Arak, Iran; 7 Student Research Committee, School of Medicine, Arak University of Medical Sciences, Arak, Iran; 8 Department of Health Services Research, National Institute for Health Research Collaboration for Leadership in Applied Health Research and Care North West Coast (NIHR CLAHRC NWC), Institute of Psychology, Health, and Society, University of Liverpool, Liverpool, UK

**Keywords:** Abuse, Functional abdominal pain, Child, Case-control study

## Abstract

**Background:**

Gastrointestinal (GI) disorders are ranked first amongst medical diseases as a trigger of requests for mental health counselling. Child abuse has been regarded as one of the main causes of the development of functional abdominal pain (FAP) in children. This study aimed, therefore, to compare the prevalence of child abuse experience among two groups of patients with and without FAP.

**Methods:**

A case-control study of children in Arak, Iran, in which experience of child abuse was compared in children with (*n =* 100) and without functional abdominal pain (*n =* 100). Three categories of child abuse - emotional abuse, physical abuse, and neglect - were assessed using the Child Abuse Questionnaire. The data were analyzed using Stata software.

**Results:**

After adjusting for potential confounders, there were group differences in emotional abuse (96% vs. 81%, aOR = 5.13, 95% CI: 1.3–20.3, *p* = 0.017), neglect (28% vs. 8%, aOR = 4.27, 95% CI: 1.8–11.8, *p* = 0.001) and total child abuse score (98% vs. 84%, aOR = 8.2, 95% CI: 1.5–43.8, *p* = 0.014) but not in physical abuse (57% vs. 46%, aOR = 1.47, 95% CI: 0.81–2.60, *p* = 0.728).

**Conclusions:**

As the prevalence of child abuse is higher in patients with FAP, child abuse appears to be related to the occurrence of FAP in children. However, the results of this study cannot be generalized to Iranian society generally and further longitudinal studies are recommended.

## Background

Chronic or functional abdominal pain (FAP) is one of the most common physical complaints in children [1–5], causing prolonged absence from kindergarten and school, parental anxiety, frequent medical referrals and numerous diagnostic procedures that impose significant costs on the family and the health system. The origin of abdominal pain can be organic or functional. Functional gastrointestinal (GI) diseases are the most common cause of chronic abdominal pain in children. FAP is a description of symptoms rather than a specific diagnosis and is defined as abdominal pain in children without serologic, mucosal, radiographic or structural evidence of disease [6, 7]. The origin of FAP is not known, although it is thought to be multifactorial, encompassing the intestinal nervous system, central nervous system (CNS), increased visceral sensitivity, psychological factors and abnormal responses to normal and abnormal physiological stimulation [2, 8]. The worldwide prevalence of FAP was 13.5% (range: 1.6–41.2%), with the highest prevalence in South America and Asia (16.8 and 16.5%, respectively) [4].

Social, psychological factors and life events (such as the death of parents in childhood, divorce or separation of parents, change of school, examinations, etc.) have been cited as possible reasons for FAP in children [2, 5, 9]. Some studies suggested that these factors are associated with GI motility [2] and also there is some evidence regarding the association of anxiety and depressive disorders with irritable bowel syndrome and ulcerative colitis [10]. In addition, caregiver anxiety may contribute to GI motility [2].

Adverse childhood experiences (ACEs), also recognized as early adverse life events (EALs), that include counting physical, emotional, and sexual events and neglect are common in children and have long-term effects on mental and physical health [11, 12]. ACEs are of the most severe and common source of stress [13]. Previous studies have shown that ACEs can be associated with numerous problems in later ages including mental health, substance abuse, poor physical health, poor functional outcomes [14], hallucination [15], depression, cardiovascular diseases, diabetes, cancer, premature mortality [16], irritable bowel syndrome and gastrointestinal symptom severity [17]. Emotional abuse, physical abuse, and neglect are considered as ACEs and their negative consequences are not enough studied in Iranian population and need further studies. In this study, we aimed to examine the ACEs association with FAP.

Previous studies suggested that there is a link between FAP and child abuse in any form so that FAP is more prevalent in those who experienced child abuse [4, 18, 19]. In a study, Devanarayana N et al. revealed that the frequency of AFP was higher in those who experienced sexual abuse (35.3% vs. 17.3%), emotional abuse (27.4% vs. 16.9%) and physical abuse (19.7% vs. 12.6%) [18]. Also, in another study, the findings suggested that there was an association between irritable bowel syndrome in adults and a history of physical, emotional, and sexual abuse [20]. On the contrary, some studies have reported that there is no difference between an abused and non-abused child in terms of functional disorders [21].

Demonstrating the presence of a relationship between FAP and child abuse would encourage healthcare providers to develop and implement programs to enhance families’ awareness of and ability to address factors related to chronic abdominal pain in children to reduce its incidence. Given the extant evidence on the role of stress in functional GI disorders, stress, anxiety and other psychological consequences of child abuse and neglect, lack of valid information, and the inconsistency between the findings of different studies, this study aimed to compare the prevalence of emotional abuse, physical abuse, and neglect experience among two groups of patients with and without FAP. The most important hypothesis in this study was that the prevalence of child abuse was higher among children with FAP. It was particularly hypothesized that the prevalence of emotional and physical abuse would be significantly associated with FAP.

## Methods

We used STROBE (The Strengthening the Reporting of Observational Studies in Epidemiology) as a guideline to design the study and report the findings [22]. This case-control study was designed to compare the prevalence of abuse in children with (as the case group) and without FAP (as the control group). The case group comprised of 100 children of both genders, who were 5–15 years old and were referred to the pediatric gastroenterology clinic of Amir-Kabir Hospital in 2016. The FAP cases were diagnosed by a pediatric gastroenterology specialist. The control group comprised of 100 children without FAP of both genders who were 5–15 years old, from the same population as the case group. Amir-Kabir Hospital is the most important referral hospital for children with different diseases and problems in Arak, Iran, and all children with different problems are referred to this hospital. Thus, both the case and control groups were selected from the same source population. The control group consisted of children referred to the outpatient clinic of the hospital for reasons other than gastrointestinal problems and they had no known chronic disease and did not take any particular medication.

To select the case group, children who showed signs of FAP were examined and if the diagnosis was confirmed by a pediatric gastroenterologist based on the Rome III criteria, a child abuse questionnaire and a demographic questionnaire were filled out.

The inclusion criteria were as follows: (1) diagnosis of FAP by a pediatric gastroenterologist (case group only); (2) being over 5 years old; (3) no history of other physical and psychiatric disorder in the child or family (parents were asked to reveal the history and its determination was self-explanatory); and (4) no mental retardation based on the interview. If the child or guardian does not want to continue the study, they were excluded.

The data collection tool was a child abuse validated questionnaire in Farsi. This questionnaire was based on the questions derived from the world-class validated ISPCAN Child Abuse Screening Tool Children’s Version (ICAST-C) and Juvenile Victimisation Questionnaire (JVQ) and was developed by a group of scholars at Tehran University of Medical Sciences [23]. Two criteria were used to evaluate the reliability of the questionnaire: intra-class correlation coefficient (ICC = 0.95) and Cronbach’s alpha (α = 0.92). The questionnaire assessed three broad categories of child abuse: neglect and physical and psychological maltreatment. Due to the sensitivity surrounding child sex abuse and the lack of compatibility with the concepts used in Iranian culture, the questions dealing with sex abuse were removed from the questionnaire. The questionnaire comprised 26 questions, distributed as follows: psycho-emotional abuse, 10 questions; physical abuse, 10 questions; and neglect, 6 questions. The response options for all questions were ‘No, never’, ‘Yes, sometimes/ a little’ and ‘Yes, always/ a lot’.

Children who responded yes at least to one of the questions in all three categories were placed to the group of abused children [24, 25]. The questionnaire was administered by the research executive, who gave all subjects the same information beforehand. All the subjects’ information remained confidential throughout the study.

All data collection was carried out by people blind to the group status (case vs. control) of the subjects to minimize bias.

Based on a type-one error rate of 0.05, study power of 0.80 and rates of physical child abuse of 20% in control group [26], to find a 20% difference between the two groups according to expert opinion (prevalence in the case group is equal to 40%), we calculated that approximately 100 subjects per group would be required.

### Ethical consideration

As children are not capable of providing ethical consent to participation the parents or legal guardians of subjects provided written informed consent on their behalf. The children were also asked verbally if they were willing to participate in the study. All stages of research were conducted following the Declaration of Helsinki and the Ethical Statements of the Ethics Committee of Arak University of Medical Sciences. This study was approved by the Ethics Committee of Arak University of Medical Sciences (Ethical code: IR.ARAKMU.REC.1394.293).

### Statistical analysis

To describe the data, qualitative variables are presented as frequencies and percentages and quantitative variables are presented as means and standard deviations. To compare the interested variables among case and control groups, independent-samples *t*-tests, likelihood ratio chi-square tests and logistic regression (adjusted for confounders) were used and crude and adjusted ORs were reported. In this study, FAP was considered as a dependent variable and any kind of child abuse was considered as an independent variable. In some analyses, because of sparse data bias or small sample bias, the estimated confidence intervals were too wide. In cases of sparse data bias, the penalized log-likelihood method was used to handle the wide confidence intervals (using the firthlogit command in Stata) [27, 28]. All the analyses were carried out with the Stata statistical software (Stata Corp LP, College Station, TX Stata).

## Results

In this study, to select the case group, 650 patients who were referred to the pediatric gastroenterology clinic were initially recruited and assessed for eligibility. Five hundred fifty patients were excluded because they did not meet the inclusion criteria or declined to participate. To select the control group, 220 patients who were referred to the pediatric clinic were assessed for eligibility and 100 participants were selected the control group (120 participants screened for the control group was excluded because they did not meet the inclusion criteria or refused to participate in the study). All the required data were collected from both groups and were included in the final analysis.

There was no group difference in terms of age (*p* = 0.902; the mean age of the cases was 8.19 years (*SD =* 2.36) and the mean age of the controls was 8.15 years (*SD* = 2.23)). Gender composition of the groups was also similar (cases: 64% female; controls: 53% female; *p* 0.114), as was the BMI distribution (*p* = 0.782). Descriptive features of demographic variables - i.e. age, gender, maternal occupation, type of water used by family, failure to thrive (FTT), birth weight, gestational age, type of feeding (breast; formula), mother’s number of pregnancies, place of residence, history of surgery, school level, and use of school toilets - are presented in Table 1. As shown in Table 1, there was a group difference in terms of smoking (*p =* 0.015), and a history of constipation was more reported in the cases than control (43% vs. 4%; *p =* 0.001).

**Table 1:** Distribution of demographic variables in case and control groups

Variables		Control group	Case group	*P* value
Age	Mean (S.D)	8.15 (2.23)	8.19 (2.36)	0.902
Sex	Girl	53 (53%)	64 (64%)	0.114
	Boy	47 (47%)	36 (36%)	
Mother’s occupation	Employed	8 (8%)	17 (17%)	0.135
	Housewife	92 (92%)	83 (83%)	
Type of consumed water	Tap water	78 (78%)	77 (77%)	0.866
	Household water treatment facilities	22 (22%)	23 (23%)	
Failure to thrive (FTT)	Yes	0	1 (1%)	0.316
	No	100 (100%)	99 (99%)	
History of feeding	Breast feeding	89 (89%)	92 (92%)	0.469
	Formula feeding	11 (11%)	8 (8%)	
Birth weight	< 1500	2 (2%)	0	0.264
	1500–2499	6 (6%)	9 (9%)	
	2500–3999	90 (90%)	86 (86%)	
	> 4000	2 (2%)	5 (5%)	
Gestational age	< 37 weeks	5 (5%)	5 (5%)	0.605
	37–42 weeks	94 (94%)	95 (95%)	
	> 42 weeks	1 (1%)	0	
Residency	Rural area	7 (7%)	12 (12%)	0.228
	Urban area	93 (93%)	88 (88%)	
Smoking Status	Smoker	1 (1%)	2 (2%)	0.015
	Exposed	16 (16%)	33 (33%)	
	None	83 (83%)	65 (65%)	
Number of mother’s pregnancies	First	57 (57%)	54 (54%)	0.790
	Second	33 (33%)	33 (33%)	
	Third or more	10 (10%)	13 (13%)	
Going to school or kindergarten	Yes	97 (97%)	91 (91%)	0.074
	No	3 (3%)	9 (9%)	
Using school toilets	Yes	60 (60%)	49 (49%)	0.118
	No	40 (40%)	51 (51%)	
History of surgery	Yes	6 (6%)	11 (11%)	0.205
	No	94 (94%)	89 (89%)	
History of constipation	Yes	4 (4%)	43 (43%)	0.001
	No	96 (96%)	57 (57%)	

As shown in Table 2, emotional abuse (96% vs. 81%), neglect (28% vs. 8%) and total abuse (98% vs. 84%) were more frequent in the case group, but the frequency of physical abuse was similar in the two groups (57% vs. 46%, *p =* 0.120). After adjusting for potential confounders (age, sex, smoking status and history of constipation) there were group differences in emotional abuse (case: 96%, control: 81%, aOR = 5.13, 95% CI: 1.3–20.3, *p* = 0.017), neglect (case: 28%, control: 8%, aOR = 4.27, 95% CI: 1.8–11.8, *p* = 0.001) and total score (case: 98%, control: 84%, aOR = 8.2, 95% CI: 1.5–43.8, *p* = 0.014), but not in physical abuse (case: 57%, control: 46%, aOR = 1.47, 95% CI: 0.81–2.60, *p* = 0.728).

**Table 2:** Distribution of child abuse and its dimensions in case and control groups

Child abuse		Control group	Case group	z	Crude OR (95% CI)	z	Adjusted OR^a^ (95% CI)
Emotional	No	19 (19%)	4 (4%)	–	–	–	–
	Yes	81 (81%)	96 (96%)	3.01	5.13 (1.8–14.9)	2.39	5.22 (1.3–20.3)
Physical	No	54 (54%)	43 (43%)	–	–	–	–
	Yes	46 (46%)	57 (57%)	1.55	1.5 (0.88–2.7)	0.35	1.12 (0.57–2.2)
Neglect	No	92 (92%)	72 (72%)	–	–	–	–
	Yes	8 (8%)	28 (28%)	3.45	4.27 (1.9–9.8)	3.24	4.7 (1.8–11.8)
Total	No	16 (16%)	2 (2%)	–	–	–	–
	Yes	84 (84%)	98 (98%)	2.94	7.7 (1.97–30)	2.46	8.2 (1.5–43.8)

^a^Adjusted for age, sex, smoking status and history of constipation

## Discussion

This study was conducted to investigate the history of abuse in children over the age of 5 with and without FAP. We found that after adjusting for confounder variables, negligence and emotional maltreatment were more prevalent in children with FAP; overall child abuse rate in the categories investigated (emotional abuse, physical abuse, and neglect) was also more frequent in the cases than controls. However, the frequency of physical abuse was similar in the two groups (case: 57%; control: 46%).

Since the results of our study showed that the prevalence of child abuse is significantly higher in patients with FAP, it is important to pay attention to psychological problems and stressful problems in these patients.

The findings of our study revealed that the frequency of emotional abuse (case: 96%; control: 81%) and any kind of abuse (case: 98%; control: 84%) was very high in our sample. As with the findings of our study, other studies have also shown that ACEs can have some adverse effects on mental and physical health [11, 12] as they are associated with poor mental and physical health, poor functional outcomes, hallucinations, depression, cardiovascular disease, diabetes, cancer, irritable bowel syndrome and gastrointestinal symptom severity [14–17]. Accordingly, our findings from a sample from a developing country are consistent with reported findings across the globe. Moreover, our findings suggested that child abuse, after adjusting for confounders, is associated with a higher odd of FAP, just like some global reports.

A few studies are conducted to assess the relationship between child abuse and FAP, and still, this relationship remains unclear and more studies are needed. In some studies, the relationship between child abuse and irritable bowel syndrome [29–31], and also abdominal pain, nausea, vomiting, and unexplained gastrointestinal symptoms were assessed [9]. Boey et al. [32] in their study concluded that abdominal pain is linked with stressful life events in children. Since child abuse, especially physical and sexual abuse, is associated with increased stress, it can lead to some diseases such as gastrointestinal problems. The findings of our study are in line with this and confirm the link between child abuse and FAP.

A cross-sectional study by Devanarayana et al. [33] investigated the relationship between functional GI disorder and exposure to child abuse in teenagers and found that the prevalence of functional GI diseases was higher in those who had experienced sexual, emotional and physical abuse in childhood than those who had not. Our results on emotional abuse and overall abuse are consistent with this earlier study, but our findings on physical abuse are not. Devanarayana et al. study [33] was a cross-sectional study on school-aged teenagers (13–18 years old), with a mean age of 14, while our study was performed on children aged 5 to 15, with an average of 8, and perhaps one of the reasons for the inconsistency in the relationship between physical abuse and FAP is the participants’ age as the prevalence of physical abuse is lower at early ages.

In another study by Devanarayana et al. [34] the authors investigated the relationship between harmful life events and abdominal pain associated with a functional gastrointestinal disorder. This study was consistent with our findings in that it found that exposure to adverse life events increased the risk of developing functional GI disorders in children. Exposure to adverse life events in childhood negatively affected the quality of life, specifically the health and physical wellbeing, of children with functional GI disorders. The findings of our study are in line with this study. Child abuse is likely to lead to an increase in diseases such as FAP through increased stress. Previous studies have confirmed that some fraction of chronic visceral pain in the gastrointestinal system is induced by stress [35].

Another multisite, longitudinal study of physical, emotional and sexual abuse and neglect among children [9] produced results that are consistent with ours, demonstrating that young people who had been subjected to maltreatment had a higher risk of developing unexplained GI symptoms. The authors suggested that the relationship between abuse and unexplained GI symptoms was partly mediated by psychological stress.

A systematic review of the impact of childhood maltreatment on quality of life [36] in which 19 articles were included, concluded that there is a negative association between child maltreatment and health-related quality of life. Our study is consistent with this conclusion, showing that the history of childhood maltreatment is related to FAP.

Previous studies have reported that rates of exposure to physical and sexual abuse are higher in people with irritable bowel syndrome than healthy people and people with organic GI diseases. Hobbis et al. [37] investigated the hypothesis that being a survivor of abusive behavior makes people susceptible to functional GI diseases and could not confirm it. This finding is inconsistent with our results. The reason for this inconsistency in the findings may be due to the smaller sample size in the study by Hobbis et al. [37]. However, they compared the prevalence of child abuse among three groups of patients including patients with idiopathic constipation, irritable bowel syndrome and Crohn’s disease with non-patient control subjects.

Our study has shown that smoking status and history of constipation can be related to FAP. Further research into these results is recommended.

One of the important limitations of our study is that in some analyses the 95% confidence intervals were too wide. We tried to address this problem by using advanced statistical methods (penalized log-likelihood method). Another limitation was the cultural restrictions in examining sexual abuse. Besides, the validity of the answers - especially those given by children under 12 years old - to questions about parental child abuse can be questionable.

In terms of the generalizability of findings, it should be highlighted that as our study was conducted in a developing country with a relatively high prevalence rate of child abuse, it is recommended to generalize the findings with more caution. Also, because the data was collected from a single-center study, generalizability to other patient populations is questionable. Besides, the hospital in which this study was conducted was a tertiary hospital with governmental tariffs and patients with lower/moderate socioeconomic status are more likely to be referred to these hospitals in Iran. Therefore; the participants in our study do not represent the whole patients.

## Conclusion

In summary, it can be concluded from our findings that the prevalence of child abuse in patients with FAP is relatively high and child abuse, particularly neglect and emotional maltreatment, is associated with FAP in Iranian children. It means that the risk of FAP in those with a history of child abuse is significantly high. It is therefore recommended that such patients should be referred to psychiatric visits. Further research in a larger sample, investigating all aspects of child abuse, is recommended to obtain more accurate and generalizable results. However, due to the limitations mentioned above, our findings need more caution in the time of interpretation.

## Data Availability

The datasets used and analyzed during the current study are available to be collected from the corresponding author on reasonable request.

## Abbreviations

FAP: Functional Abdominal Pain
OR: Odds Ratio
aOR: Adjusted Odds Ratio
GI: Gastrointestinal
FTT: Failure to thrive
CI: Confidence Interval
BMI: Body Mass Index
